# Is fibrodysplasia ossificans progressiva an interleukin-1 driven auto-inflammatory syndrome?

**DOI:** 10.1186/s12969-019-0386-6

**Published:** 2019-12-21

**Authors:** Ruby Haviv, Veronica Moshe, Fabrizio De Benedetti, Giusi Prencipe, Noa Rabinowicz, Yosef Uziel

**Affiliations:** 1 Pediatric Rheumatology Unit, Department of Pediatrics, Meir Medical Center, 59 Tchernichovsky St., 4428164 Kfar Saba, Israel; 2 Sackler Faculty of Medicine, Tel Aviv University, Tel Aviv, Israel; 3 Division of Rheumatology, Bambino Gesù Children’s Hospital IRCCS, Rome, Italy

**Keywords:** Fibrodysplasia ossificans progressiva, FOP, Heterotopic ossification, Interleukin, IL-1, IL-1β, Anakinra, Canakinumab

## Abstract

**Background:**

Fibrodysplasia ossificans progressiva (FOP) is the most catastrophic form of heterotopic ossification, due to ongoing intracellular signaling through the bone morphogenic protein pathway. The paroxysmal appearance of inflammatory lumps and elevated inflammatory markers during flares, suggest that FOP is an auto-inflammatory disease. Based on evidence, demonstrating a role for interleukin-1β (IL-1β) in other forms of heterotopic ossification, we hypothesized that treating FOP patients with anti-IL-1 agents could help lower the rate of FOP paroxysms and/or limit the symptoms and residual lesions.

**Case presentation:**

A 13.5-year-old Arab boy was diagnosed with FOP. Treatment with anti-inflammatory drugs did not change the disease course. New lumps appeared in a rate of approximately one every 8 days. Treatment with the anti-IL-1 agents anakinra and canakinumab resulted in significantly lower rate of paroxysms (every 22–25 days, of which almost all involved only 2 existing lumps), as well as shorter duration. High levels of IL-1β were found in the patient’s plasma samples, collected during a paroxysm that appeared 8 weeks after the last canakinumab dose. In contrast, IL-1β plasma levels were undetectable in the previous three plasma samples, obtained while he was treated with anti-IL-1 agents.

**Conclusions:**

Our data demonstrate the efficacy of anti-IL-1 agents in the treatment of a patient with FOP. Results showing the marked increase in IL-1β plasma levels during a paroxysm support a role for IL-1β in the pathogenesis of FOP and further provide the rationale for the use of anti-IL-1 agents in FOP treatment.

## Background

Fibrodysplasia ossificans progressiva (FOP) is the most catastrophic form of heterotopic ossification (HO). This rare genetic disease (1 in 1,360,000–2,000,000) [1] is caused by mutations in the *ACVR1/ALK2* gene, encoding the type 1 Activin A receptor, which is part of the heterodimeric type I bone morphogenic protein (BMP) receptor. R206H missense gain-of-function is the most frequent mutation, and is located at the end of the highly conserved glycine-serine region of the cytoplasmic domain of the receptor [2], adjacent to the protein kinase domain. Gain of function mutations in *ACVR1* cause ongoing intra-cellular signaling of the BMP pathway (through phosphorylation of Smad1/5/8), which alters cellular fate and induces undifferentiated mesenchymal cells to form cartilage, and later on leads to complete ossification of muscle, as well as subcutaneous and other mesenchymal tissues.

The heterotopic bone continues to expand and even remodels itself through an Activin A-dependent process [3, 4]. Activin A (as other Activins) is also known to have an inhibitory role, as it competes with BMP in binding to its receptor, but does not induce downstream phosphorylation of the transcription factors Smad1/5/8 [4].

Clinically, painful, soft tissue swellings usually start appearing during the first decade of life, and 95 percent of FOP patients experience their first “paroxysm” before the age of 15 years. However, a typical, bilateral deformity of the hallux can be noted at birth in about 80% of patients [5].

Currently, there is no established, effective treatment for FOP. Of the few anti-inflammatory therapies reported, such as anti-leukotrienes, non-steroidal anti-inflammatory drugs, mast-cell stabilizers [6] and sirolimus [7], none had a major effect on disease progression. When lumps appear, high dose corticosteroids (either oral prednisone 2 mg/kg/day or intravenous methylprednisolone pulse), along with a bisphosphonate infusion, are used [6]. A few specific drugs are “in the pipeline” (Regenron’s garetosmab, an anti-Activin A antibody and Clementia’s palovarotene, a retinoic acid receptor-gamma agonist) [7], but these are still unavailable for prescription. Anti-tumor necrosis factor α agents were not successful in treating the disease (personal communication).

Average life expectancy is around 45 years. By the third decade of life, most FOP patients are wheelchair-bound [6]. A main cause of morbidity is related to ankylosis of the temporomandibular joints and the most common cause of mortality is thoracic insufficiency syndrome [5, 7–9].

The recurrent paroxysmal appearance of inflammatory lumps (tender, localized swellings, with erythematous skin superficially, which partially react to anti-inflammatory agents), accompanied by elevated inflammatory markers during flares, may suggest that FOP is an auto-inflammatory disease. The episodic formation of bone, often following a trivial injury, suggests that innate immune-related triggers induce tissue transformation through the BMP pathway [10]. Moreover, interleukin-1β (IL-1β), a well-known mediator of the innate immune system, has been linked to HO and mineralization in human bone marrow-derived mesenchymal stem cell cultures [11–13]. We hypothesized that treating a FOP patient with anti-IL-1 agents could help ameliorate the progression of this devastating disease, by slowing the rate of paroxysms, and/or limiting the symptoms and residual lesions. We report our experience.

## Case presentation

**Fig. 1:**
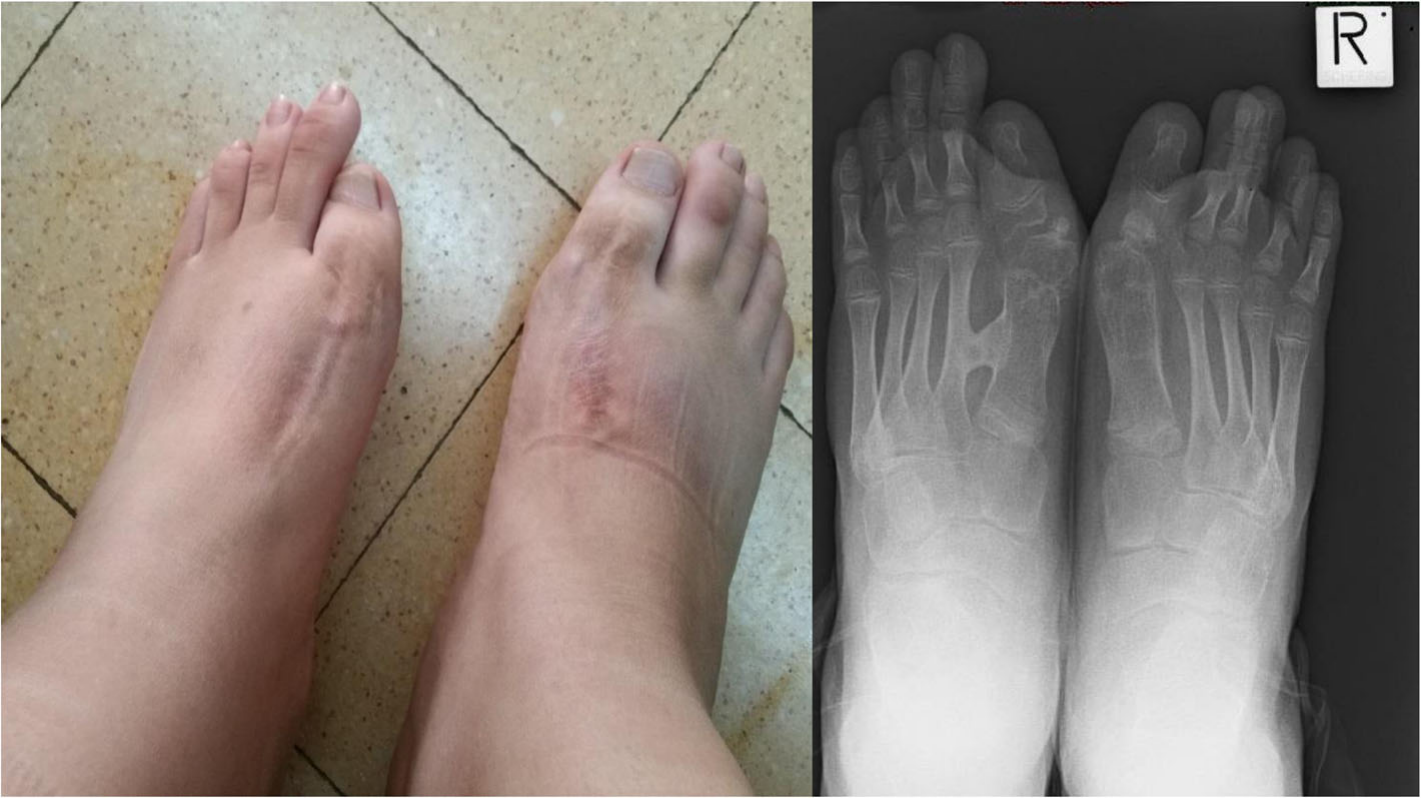
The patient’s halluces after an osteotomy procedure. Note the bony mass between the first and second metatarsal bones on the left foot

A 13.5-year-old, Muslim Arab boy was diagnosed clinically with FOP. Diagnosis was confirmed after genetic testing (the typical R206H mutation in the ACVR1/ALK2 gene was found). When first examined, he already had asymmetrical shoulder positioning and limited rotation of the neck, spine and left hip. In addition, bony mass formations were palpated on his right waist, medial to his right scapula and left paravertebral region. He also had non-rigid, non-tender, warm swellings within the sternocleidomastoid muscles, bilaterally. His halluces were abnormally short and wide. He had undergone an osteotomy when he was 7-years-old, to straighten their congenital valgus position (Fig. 1).

Laboratory tests results showed mildly elevated inflammatory markers, including CRP (approximately 3 mg/dL, normal values 0–0.5 mg/dL), mild leukocytosis with moderate neutrophilia and mild microcytic anemia.

**Fig. 2:**
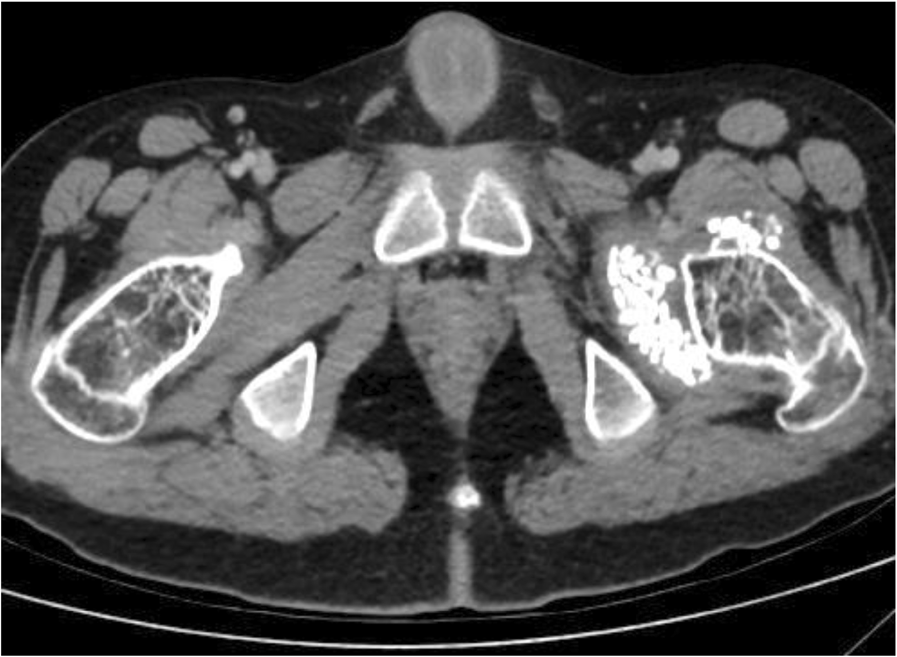
“Chondrocalcinosis” of the left hip joint capsule and generalized osteopenia on total body CT

**Fig. 3:**
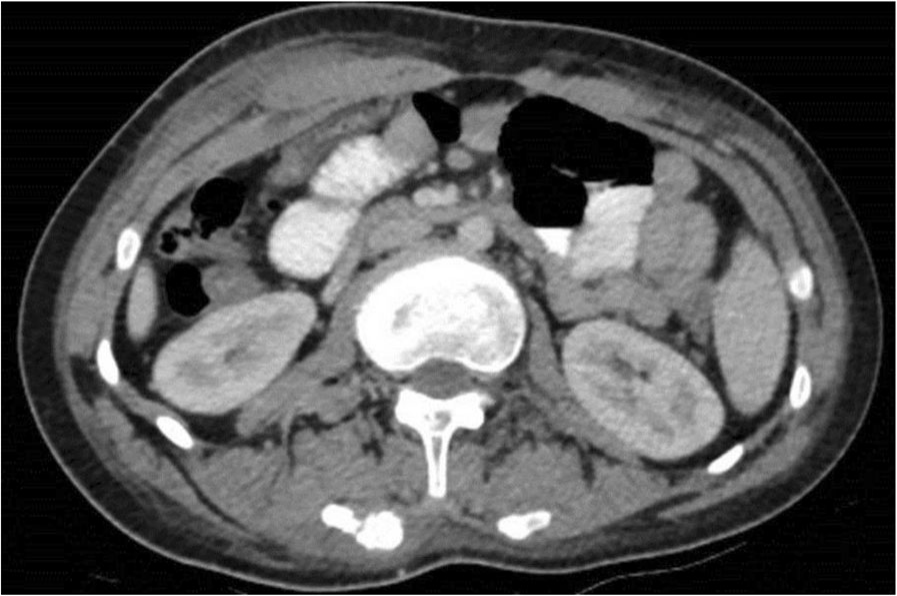
Coarse “calcifications” within lumbar paravertebral muscles on total body CT

**Fig. 4:**
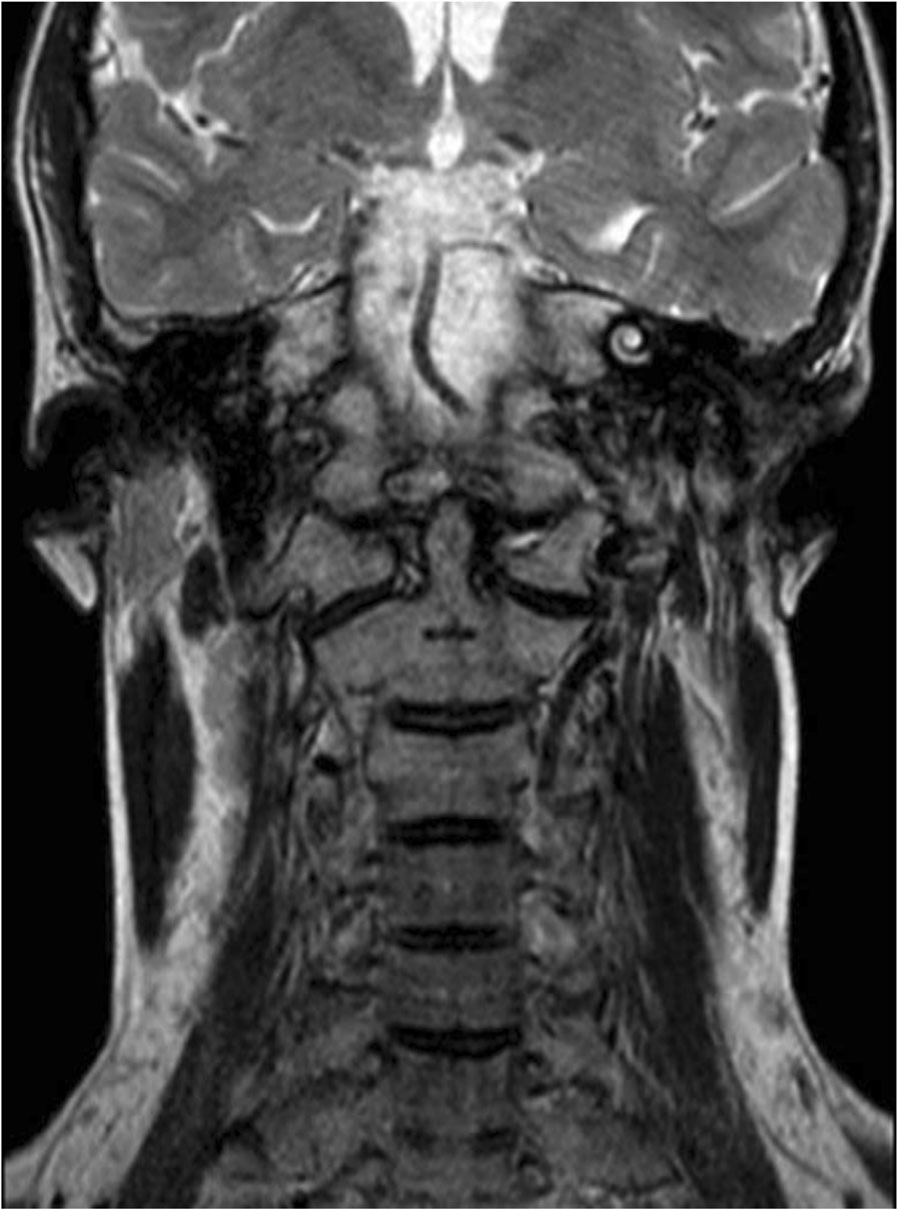
Non-homogenously edematous sternocleidomastoid muscles and infiltrated fat on the cervical spine MRI scan

Total body CT revealed generalized osteopenia, “chondrocalcinosis” of the left hip joint capsule, thickened fascia and infiltrated fat and anteversion of the femoral head (Fig. 2). Coarse lumbar paravertebral calcifications within muscles were also seen (Fig. 3). MRI of the cervical spine revealed non-homogenously edematous sternocleidomastoid muscles and thickening of the strap muscles and the muscles around his right scapula, thickening of the submental fascia and infiltration of subcutaneous fat (Fig. 4).

**Fig. 5:**
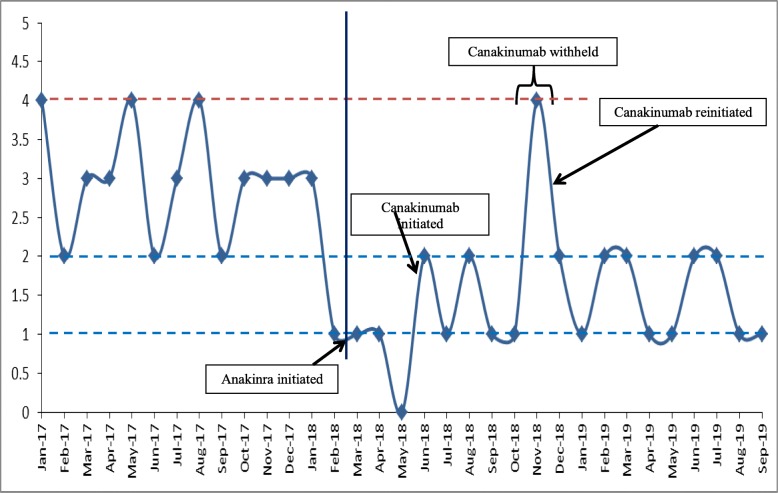
Number of FOP flares per month, based on documentation held by the patient’s family, the patient’s pediatrician and the rheumatology unit. Note the marked change in the mean number of flares, just after the initiation of anti-IL-1 treatment (between the dashed blue lines), and a temporary elevation in flare frequency while canakinumab was withheld

Treatment with high-dose corticosteroids, pamidronate infusions, and daily celecoxib and monteleukast did not change the course of the disease, and new lumps continued to appear at a rate of approximately one new lump every 8 days. Anakinra treatment was initiated (100 mg/day; patient’s weight was over 100 kg). After marked improvement was noted over a 2-month period, he was treated with canakinumab 300 mg, every 4 weeks, for 5 more months. Markedly lower rate and duration of paroxysms was documented (Fig. 5 clearly shows a reduction from 3 to 4 to 1–2 flares per month, one every 22–25 days, of which almost all involved only 2 existing lumps – see more below), with shorter courses of methylprednisolone during flares (mostly 1–2 doses, instead of 3). Moreover, lumps were markedly smaller and softer.

Canakinumab treatment was then withheld for 8 weeks. During this time, lumps appeared more frequently. He experienced one flare during the 36 days following the fifth canakinumab dose, but at least 4 more between day 37 and day 54, with the appearance of new lumps on his torso and neck, and especially a large lump below his left scapula. Canakinumab treatment was then reinitiated.

### Cytokine measurements

Plasma IL-1β, IL-6 and IL-18 levels were measured in samples collected from the patient, and from controls, by enzyme-linked immunosorbent assay, using the Human IL-1b /IL-1F2 Quantikine High Sensitivity ELISA Kit (R&D System), the Human IL-6 Quantikine High Sensitivity ELISA Kit (R&D System) and the Human IL-18 Elisa kit (MBL corporation), according to the manufacturer’s instructions.

**Fig. 6:**
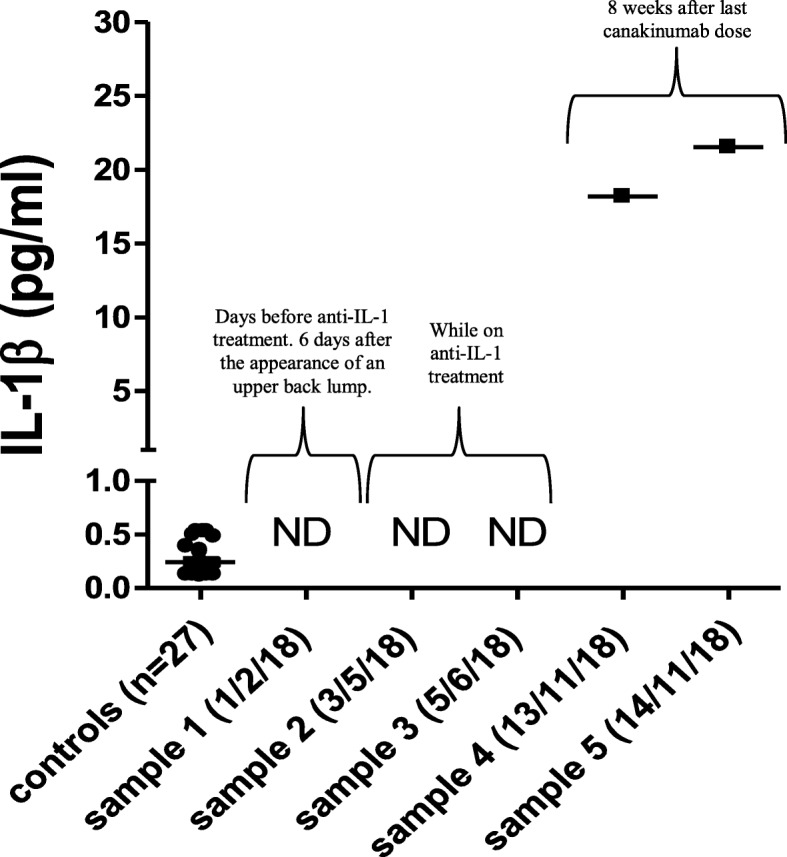
High levels of IL-1β (up to 21.52 pg/ml) were found in the patient’s plasma (13–14/11/2018) during the appearance of a large new lump below his left scapula, after canakinumab treatment was withheld for 8 weeks. In contrast, undetectable (ND) levels of IL-1β (< 0.125 pg/ml) were observed in the previous three plasma samples obtained during treatment with anakinra (03/05/2018) or canakinumab (05/06/2018), and without temporal proximity to a new lump (01/02/2018)

**Fig. 7:**
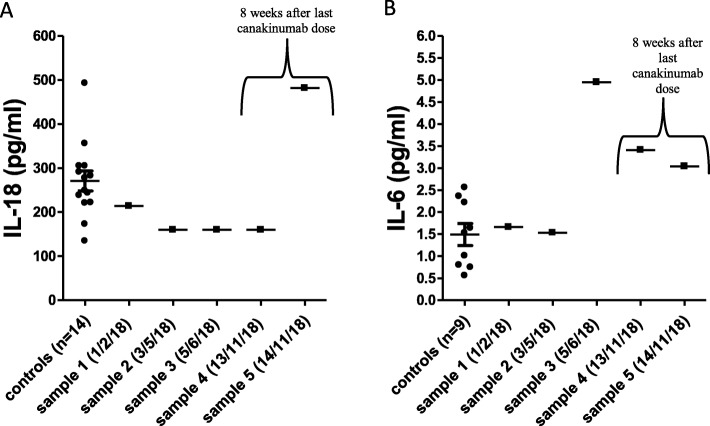
IL-18 (**a**) and IL-6 (**b**) plasma levels, measured before, during and after withholding treatment, were comparable or slightly higher than those observed in healthy controls

Notably, while unmeasurable levels of IL-1β (< 0.125 pg/ml) were found in the three plasma samples obtained from the patient during treatment with anakinra or canakinumab, high levels of IL-1β (up to 21.52 pg/ml, about 90-fold higher compared to average levels measured in healthy controls) were found in the patient’s plasma samples collected during the flare (Fig. 6). In contrast, IL-18 and IL-6 plasma levels, measured before, during and after withholding treatment, were comparable or slightly higher than those observed in healthy controls (Fig. 7a, b).

**Fig. 8:**
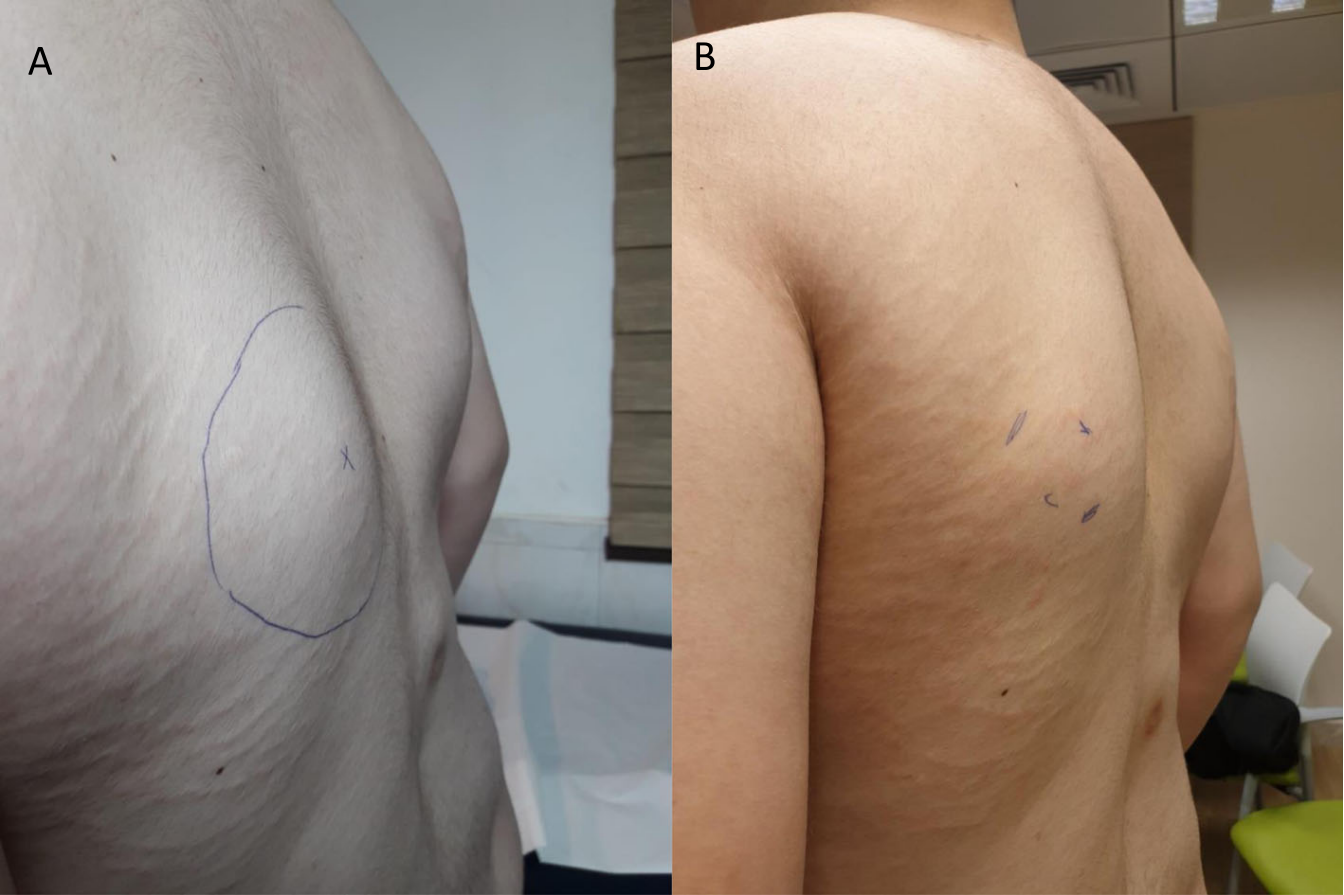
Left scapular lump decreased from 4.5 × 4.5 cm (**a**) to 3.5 × 2.5 cm (**b**) within 2 weeks after subcutaneous injection of canakinumab and without corticosteroids

The patient has been treated with canakinumab approximately 3.5 mg/kg/month for more than 16 months, with a relatively low flare rate of approximately one every 25 days (Fig. 5). The lumps involved in almost all of these flares are the same: at the left scapular base and within the right sternocleidomastoid muscle. We documented a decrease in the size of the left scapular lump from 4.5 × 4.5 cm to 3.5 × 2.5 cm within 2 weeks after subcutaneous injection of canakinumab, without the addition of corticosteroids (Fig. 8).

## Discussion

To the best of our knowledge, this is the first time anti-IL-1 agents have been shown to ameliorate the natural progression of FOP. In addition, plasma IL-1β, IL-18 and IL-6 levels in longitudinal samples, collected from a FOP patient, show that IL-β levels, but not IL-18 and IL-6 levels, increased significantly during flares.

Activins and BMPs are expressed by innate immune system cells during inflammation [4]. The linkage between FOP, heterotopic bone formation and inflammation was observed long ago, and macrophages derived from FOP patients have been found in a pro-inflammatory state, with increased expression of nuclear factor kappa B (NF-κB) and mitogen-activated protein kinase (MAPK) [10, 14]. Elevated IL-3, IL-12p70 (the active heterodimer form), IL-6, and other inflammatory factors have been linked to HO, related to combat wounds and high-energy trauma [15, 16].

Although IL-1β has been studied minimally in the context of FOP, it has been linked to HO through several studies. Mahy and Urist reported that recombinant IL-1β enhanced a process of BMP-induced HO, and this effect was completely eliminated by administration of antibodies against IL-1 [11]. Ferreira and colleagues reported that IL-1β and TNF-α strongly induced mineralization in 8 of 10 cultures of human bone marrow-derived mesenchymal stem cells. The IL-1β were mediated via the MAPK, rather than the NF-κB signaling pathway, despite suppression of osteoblastic differentiation. This study suggested that suppressed expression of the ectonucleotide pyrophosphatase/phosphodiesterase-1 gene (*ENPP1*) could be one of the causes, leading to suppression of extracellular pyrophosphate (PPi) levels, which is linked to HO [12]. Ezura et al. investigated the effects of IL-1β on the mineralization of human and mouse bone marrow mesenchymal cells, by focusing on the alternative extracellular calcification mechanism through regulation of extracellular levels of inorganic phosphate (Pi), PPi, and adenosine. Characteristic suppression of the osteoblastic marker genes by IL-1β during a 20-day osteogenic culture, and reduced expression of the important transporter genes, *ANKH* (human Ank homologue) and *ENT1* (*SLC29A1*), were observed. *ANKH* and *ENT1* are the plasma membrane transporters for the byproducts of nucleotide metabolism (PPi and adenosine, respectively) and are involved in the regulatory system for HO. Extracellular Pi and PPi are well-known byproducts of nucleotide metabolism and are recognized to be very important for HO pathogenesis [13].

Using principal component analysis, Barruet and colleagues reported no significant differences in IL-1α and IL-1β levels, secreted by lipopolysaccharide (LPS)-stimulated monocytes of either control or FOP patients (during a flare), and significantly increased IL-1 receptor antagonist levels in FOP monocytes, after LPS stimulation [14]. Reviewing their report, it is important to mention that blood samples from only 4 FOP patients were used for this analysis, and higher IL-1α and IL-1β levels were measured in two of them. All 4 patients were using anti-inflammatory drugs (principally NSAIDs), one had still been using a corticosteroid 4 days before the blood sample was taken, and the other two patients were using corticosteroid 10 days before blood samples were taken.

In summary, there is growing evidence that the nature of HO in general and FOP specifically, is inflammatory, and that anti-inflammatory drugs may ameliorate this process. IL-1 has a role in this process, along with other mediators, but there are insufficient data to determine how important it is in comparison to other inflammatory cascades and mediators. Moreover, a laboratory model to link IL-1β and FOP is lacking.

In this case, a clear beneficial effect of anti-IL1 agents has been shown: a marked decrease in flare frequency, almost no appearance of new lumps and relatively smaller and softer lumps during flares. The size of the lumps was not measured by accurate imaging techniques, such as CT scans. However, measurements were done by the hands of the same physician during each clinical visit.

## Conclusion

Altogether, our data show the efficacy of treatment with anti-IL-1 agents in a FOP patient, and markedly increased levels of IL-1β during flares suggest a role for IL-1β in the pathogenesis of this disease, although it is too soon to conclude whether FOP may be included under the umbrella of auto-inflammatory syndromes. Anti-IL-1 agents may be effective in ameliorating the natural progression of FOP. Indeed, unlike the “standard” anti-inflammatory agents currently used for prophylaxis, anti-IL-1 agents were efficacious in lowering the rate and extent of heterotopic ossification in our patient. However, further exploration and international experience with other FOP patients are needed.

## Data Availability

The authors do not have permission to share the data.

## Abbreviations

BMP: Bone morphogenic protein
FOP: Fibrodysplasia ossificans progressiva
HO: Heterotopic ossification
MAPK: Mitogen-activated protein kinase
